# Tocilizumab in Systemic Juvenile Idiopathic Arthritis: Response Differs by Disease Duration at Medication Initiation and by Phenotype of Disease

**DOI:** 10.3389/fped.2021.735846

**Published:** 2021-11-08

**Authors:** Xin Yan, Wenjing Tang, Zhiyong Zhang, Yu Zhang, Chong Luo, Xuemei Tang

**Affiliations:** ^1^Department of Rheumatology and Immunology, Children's Hospital of Chongqing Medical University, Chongqing, China; ^2^Chongqing Key Laboratory of Child Infection and Immunity, Children's Hospital of Chongqing Medical University, Chongqing, China; ^3^Ministry of Education Key Laboratory of Child Development and Disorders, National Clinical Research Center for Child Health and Disorders, China Development and Critical Disorders, Children's Hospital of Chongqing Medical University, Chongqing, China

**Keywords:** systemic juvenile idiopathic arthritis, tocilizumab, clinical trial, pediatric, treatment

## Abstract

**Objective:** We performed a single-center retrospective study to determine the different efficacy of tocilizumab (TCZ) in the early and late stages and in three phenotypic subgroups (monocyclic, polycyclic, and persistent) of systemic juvenile idiopathic arthritis (sJIA).

**Methods:** Clinical and serological parameters of 77 sJIA patients treated by TCZ were collected from November 1, 2013 to May 1, 2019. Patients were grouped based on the duration group A < 6 months (*n* = 41) and group B > 6 months (*n* = 36) and divided into three phenotypes: monocyclic (*n* = 12), polycyclic (*n* = 14), and persistent (*n* = 51) course.

**Results:** At baseline, group A had pronounced ESR, fever less active arthritis than group B (*p* < 0.05). After 12 weeks of therapy, TCZ alleviated fever, ESR, CRP, and systemic-onset juvenile arthritis disease activity score-27 (sJADAS27) in both group A and group B (*p*>0.05), while the efficacy of TCZ in relieving active arthritis in group A was better than that in group B (*p*<0.05). After 1 year of TCZ therapy, it showed that patients with monocyclic phenotype had the highest clinical response rate (91.7%, odds ratio = 0, 95% CI: 24–24, *p* = 0.00), followed by the polycyclic (28.6%, odds ratio = 2.1, 95% CI: 10.5–18.8, *p* = 0.00) and the persistent course (9.8%, odds ratio = 1.2, 95% CI: 9.5–13.8, *p* = 0.00).

**Conclusion:** TCZ can quickly relieve fever and inflammation, especially when patients have less active arthritis with shorter disease duration. The long-term efficacy of TCZ is related to the phenotypes, among which the monocyclic is the best, and the persistent is the worst.

## Introduction

Systemic juvenile idiopathic arthritis (sJIA) is a systemic inflammatory disease clinically characterized by fever, lymphadenopathy, arthritis, rash, and serositis. sJIA is the most serious subtype of juvenile idiopathic arthritis (1) and accounts for 30% to 40% of all JIA in Asia (2). A significant number of patients develop severe disease and treatment-related complications such as persistent arthritis, growth delay, and osteoporosis. Serious complications which are potentially fatal including macrophage activation syndrome (MAS) occur in 10% to 15% of children with sJIA (3–5). sJIA is divided into three phenotypes: monocyclic, polycyclic, and persistent course (6, 7).

The etiology of sJIA is not fully understood; proinflammatory cytokines including interleukin (IL)-6, IL-1, and IL-18 play an important role in the pathogenesis of the disease. IL-6 mediates systemic inflammation in sJIA, leading to joint synovial hyperplasia and joint destruction (8–10). Blockade of IL-6 represents the main mechanism of sJIA treatment and prevention of potential complications (11).

In 2011, the United States and Europe successively approved tocilizumab, a humanized monoclonal antibody TCZ against the IL-6 receptor for treating children with sJIA. Due to the heterogeneity of sJIA, the clinical response of patients treated with TCZ is different; hence, it is needed to better characterize the profile of patients with sJIA who are more likely to respond to IL-6 blockade. Pacharapakornpong et al. (12) found that in the early TCZ treatment (<6 months), sJIA patients had a higher remission rate than late TCZ treatment (>6 months). In this study, we performed a single-center retrospective study to determine the different efficacy of TCZ in the early and late stages and in three phenotypic subgroups (monocyclic, polycyclic, and persistent) of sJIA. The safety profile and therapeutic effect of TCZ were observed and analyzed to provide a clinical basis for the treatment of children with sJIA.

## Materials and Methods

### Study Design and Population

We conducted a single-center retrospective study including patients with sJIA meeting the 2011 American College of Rheumatology designation criteria (13), who were starting TCZ in the Department of Rheumatology and Immunology, Chongqing Medical University, from November 1, 2013 to May 1, 2019. Patients with other rheumatic, infectious, neoplastic, and autoinflammatory diseases were excluded. Patients treated with other biological agents (e.g., infliximab and etanercept) in the previous 3 months were allowed. The enrolled patients who were in the active stage of the disease were allowed non-Steroidal anti-inflammatory drugs (NSAIDs), glucocorticoids, and disease-modifying antirheumatic drugs (DMARDs) (e.g., MTX, thalidomide, hydroxychloroquine, and leflunomide), among which the glucocorticoid dose was standardized to ≤1 mg/kg/day.

TCZ was given at a dosage of 8–12 mg/kg (12 mg/kg for body weight < 30 kg, 8 mg/kg for body weight ≥ 30 kg) with a slow intravenous infusion every 2 weeks. After 12 weeks, TCZ was given every 4 weeks and every 6 weeks after an initial 24 weeks of treatment. All children received TCZ at least six times. This study is in line with the ethical standards set by the Chinese Medical Ethics Committee, and the subject's guardians provided informed consent.

### Assessment and Outcomes

(1) The temperature, the presence of skin rash, arthritis severity, and liver and spleen lymph nodes of sJIA patients were measured before TCZ treatment, and after 2, 12, 24, and 52 weeks.

(2) Laboratory indexes of white blood cell, hemoglobin, platelet, alanine aminotransferase, aspartate aminotransferase, erythrocyte sedimentation rate (ESR), and C-reactive protein (CRP) were recorded during the TCZ treatment follow-up.

(3) The systemic-onset juvenile arthritis disease activity score-27 (14) (sJADAS-27) scoring system was used to evaluate the efficacy of TCZ treatment during the follow-up. The sJADAS-27 score includes five aspects: a physician's assessment of disease activity, parent and child's assessment of disease activity, the number of active joints, ESR, and clinical manifestations. The sum of the following five scores determines the sJADAS27 score. Assessment of disease severity: doctors, parents, and children used a 10-cm intuitive visual analog scale to evaluate disease activity, with a total score of 10 points (0 points for disease-free activity and 10 points for maximum disease activity) for the doctor score and 10 points for the parent and children score. The number of active joints: each active joint scores 1 point. Arthritis activity refers to swelling joint and limitations of joint movement due to pain or tenderness. ESR: standardized to 0 to 10 points using the formula (ESR-20)/10 (if ESR < 20 mm/h, it is converted to 0, and if ESR ≥ 120 mm/h it is converted to 10). Clinical aspects: fever, 37–38°C scores 1 point, 38–39°C scores 2 points, 39–40°C scores 3 points, >40°C, 4 points; rash scores 1 point; lymphadenopathy, liver and/or spleen swelling, serositis, anemia, hemoglobin < 90 g/l, platelets > 600 × 10^9^/l, and/or ferritin > 500 ng/ml score 1 point each. The evaluation of joints under the sJADAS27 score includes 27 joints: one cervical joint, two elbow joints, two wrist joints, six first to third metacarpophalangeal joints, 10 proximal interphalangeal joints, two hip joints, two knee joints, and two ankle joints.

(4) Based on Wallace criteria (15), therefore, no clinical activity is defined as no joint with active disease, no fever, rash, serositis, hepatosplenomegaly, or systemic lymphadenopathy caused by sJIA, no active uveitis, normal ESR/CRP level (ESR < 20 mm/H, CRP < 8 mg/l, a high ESR/CRP level not caused by sJIA is acceptable); the best possible score of disease activity is reported by a physician global assessment (such as a score of 0 on the visual analog scale); and the duration of morning stiffness is < 15 min. Clinical remission is defined as no clinically active disease for ≥ 6 months. A period of active disease is defined as one or more of the following: >1 active joint; abnormal ESR/CRP levels; a score of 0–10 on the visual analog scale; overall disease activity defined by a physician global assessment score > 0; parent and child health overall status score ≥ 0.

(5) Monocyclic: disease activity followed by a long period of remission lasting for 2 years. Polycyclic: alternating periods of disease activity and remission, manifesting as recurrent attacks. Persistent course: fever and active arthritis lasting for more than 3 months, accompanied by a significant increase in inflammatory indicators such as ESR and CRP (6, 7).

### Statistical Analysis

Statistical analysis was conducted using SPSS23.0 statistical software. The Shapiro–Wilk's test was used for checking the normality in the distribution of numeric variables. The statistical description is presented as mean and standard deviation for quantitative data conforming to the normal distribution, medians and interquartile range (IQR) for continuous variables, and number and percentages for categorical variables. Comparison between groups was analyzed by χ^2^ test, repeated measurement analysis of variance (ANOVA), and rank-sum test. Comparison of prognosis among groups was analyzed by survival analysis. *p*-value < 0.05 was considered statistically significant.

## Results

### Short-Term Efficacy of TCZ

A total of 77 sJIA patients (52 males and 25 females) with a median age of 6.3 years (2.5 ~ 12 years) and a median disease duration of 11 months (0 ~ 52 months) were enrolled at baseline. Patients were grouped based on the duration (before TCZ treatment): group A ≤ 6 months (*n* = 41) and group B > 6 months (*n* = 36) (Table 1).

**Table 1:** Baseline characteristics of patients with systemic juvenile idiopathic arthritis.

	**Group A (*n* = 41)**	**Group B (*n* = 36)**	***p*** **-value**
Male (%)	26 (63.4)	26 (72.2)	0.410
Age at diagnosis (years)	6.8(1.25–12)	5.8(2.25–13.75)	0.582
Disease duration before tocilizumab treatment (months)	0.8(0–6)	24.2(6–84)	0.000*
Number of patients who previously received DMARDs (%)	22 (53.7)	29 (80.6)	0.013*
Number of patients who previously received glucocorticoids (%)	27 (65.9)	25 (69.4)	0.737

*Data are presented as median and IQR, and number and percentages. n, number of patients; DMARDs, disease-modifying antirheumatic drugs;*

* *p < 0.05*.

At baseline, group A had pronounced ESR, fever, and less active arthritis compared to group B (*p* < 0.05). There were 40 (40/41) patients with fever in group A, 29 (29/36) in group B (Table 2). Active arthritis was mainly in the knees, ankles, wrists, and hips in both groups A and B. After 2 weeks of TCZ treatment, fever, active arthritis, sJADAS-27 score, white blood cell counts, ESR, and CRP levels had significantly relieved (*p* < 0.05) in both group A and group B. After the 12 week treatment, the effect of TCZ in relieving active arthritis in group A was better than in group B (*p* < 0.05). There was no difference between group A and group B in relieving fever, sJADAS-27 score, white blood cell count, ESR, and CRP (each *p* > 0.05), as shown in Table 2.

**Table 2:** Changes in clinical parameters after tocilizumab treatment in children with systemic juvenile idiopathic arthritis.

	**Group**	**Baseline**	**Week 2**	**Week 4**	**Week 12**	**F**	***p*** **-value**
Fever	A	40/41^1^	2/41**^a^**	4/41	6/41	0.759	0.386
	B	29/36	9/36^a^	5/36	4/36		
Number of active arthritis episodes	A	3.2 ± 3.0**^1^**	1.1 ± 1.9**^a^**	0.5 ± 1.0**^b^**	0.7 ± 1.6	6.395	0.014^*^
	B	4.4 ± 3.7	2.4 ± 2.4^a^	1.7 ± 2.0^b^	1.1 ± 1.9**^c^**		
sJADAS-27 score	A	21.7 ± 4.2	6.0 ± 4.9^a^	4.8 ± 5.3	4.9 ± 6.1	3.298	0.73
	B	21.8 ± 5.8	10.4 ± 7.0**^a^**	7.4 ± 6.4**^b^**	5.2 ± 5.2**^c^**		
WBC (× 10^9^/L)	A	19.9 ± 10.0	14.6 ± 9.0**^a^**	13.2 ± 9.1	12.7 ± 10.5	3.652	0.06
	B	15.6 ± 7.1	11.8 ± 5.6**^a^**	10.2 ± 4.2**^b^**	10.7 ± 8.0		
ESR (mm/H)	A	91.5 ± 30.6^1^	18.4 ± 20.1**^a^**	22.3 ± 36.9	26.2 ± 40.0	2.652	0.108
	B	76.8 ± 28.0	21.1 ± 21.8**^a^**	15.4 ± 23.5	13.3 ± 22.8		
CRP (mg/L)	A	78.4 ± 42.3	13.1 ± 13.1**^a^**	22.1 ± 32.5	19.1 ± 24.3	0.152	0.698
	B	89.7 ± 33.7	17.8 ± 23.9**^a^**	14.0 ± 17.6**^b^**	17.8 ± 28.5		

*Data are presented as mean ± standard deviation, and number and percentages*.

1 *Comparison between group A and group B at baseline, p < 0.05*:

a *compared with baseline, p < 0.05*;

b *compared with 2 weeks of treatment, p < 0.05;*

c *compared with 4 weeks of treatment, p < 0.05*.

* *Comparison of the effect of tocilizumab (12 weeks of treatment) in group A and group B, p < 0. 05. CRP, C-reactive protein; ESR, erythrocyte sedimentation rate; WBC, white blood cell count*.

### Long-Term Efficacy of TCZ

The treatment duration of TCZ in group A was 9.5 months (IQR 3–36) and 11.7 months (IQR 3–24) in group B. All patients were followed up for at least 24 months.

**Figure 1:**
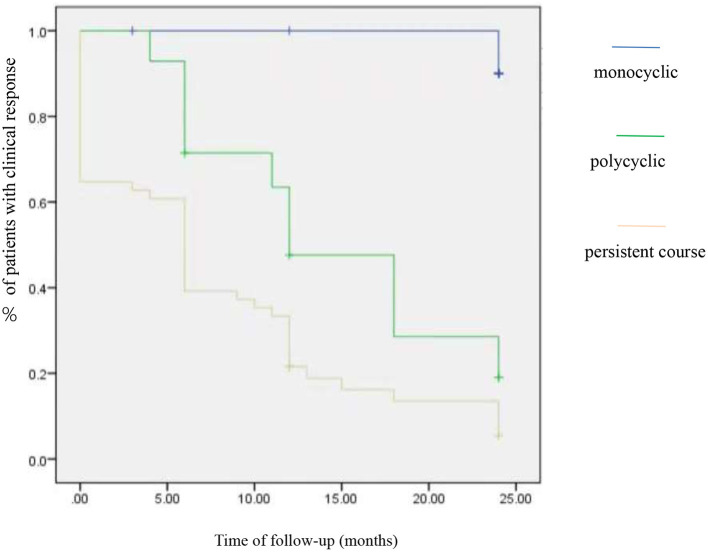
Clinical response with 12 weeks of tocilizumab treatment in different phenotypes.

At the 1 year follow-up, there was no significant difference in the proportion of patients who achieved clinical remission, no clinical activity, and clinical activity period in both group A and group B (*p* > 0.05). Three clinical phenotypes were defined as follows: monocyclic (*n* = 12), polycyclic (*n* = 14), and persistent (*n* = 51) course. A comparison of the efficacy of TCZ in patients with three phenotypes revealed significant differences in the outcome; it showed that patients with monocyclic phenotype had the highest clinical response (with no clinical manifestation and normalized inflammation parameter) rate (91.7%, odds ratio = 0, 95% CI: 24–24, *p* = 0.00), followed by the polycyclic (28.6%, odds ratio =2.1, 95% CI: 10.5–18.8, *p* = 0.00) and the persistent course (9.8%, odds ratio =1.2, 95% CI: 9.5–13.8, *p* = 0.00) (Figure 1).

A comparison of prognosis among groups was analyzed by survival analysis. All the patients were followed up for 24 months. In the initial stage of TCZ treatment, the three phenotypes of patients had good clinical responses. As time went by, the clinical response of a single course was better than polycyclic and persistent course.

## Adverse Events

This study represented 67.5 years of TCZ exposure in 77 sJIA patients; adverse events occurred in 21 patients (Table 3). Leukopenia was observed in seven patients, including one leukopenia induced by streptococcal infection, two infusion reactions characterized by fever and cold chills, and facial blushing, which occurred during the second infusion of TCZ. This was relieved by discontinuing the infusion and intravenous dexamethasone treatment and did not reoccur during later TCZ infusions. One infusion reaction occurred during the sixth TCZ infusion, presented as fever, chills, and cyanosis, and was also relieved by discontinuing the infusion and intravenous dexamethasone treatment. For this patient, TCZ treatment was terminated. Two patients experienced MAS, one at 3 months and the other at 6 months of tocilizumab treatment. Two patients had continuous active disease during tocilizumab treatment, and MAS was improved after comprehensive treatment instead of TCZ.

**Table 3:** Adverse events in patients receiving tocilizumab.

**Adverse event**	**Number**
Leukopenia	7
Elevated transaminases	5
Infusion reaction	3
Pneumonia, pulmonary consolidation	2
Septicemia	1
Streptococcal infection	1
Macrophage activation syndrome	2

## Discussion

The treatment of sJIA usually requires NSAIDs, DMARDs, and glucocorticoids (6). It is necessary to use biological agents when it is refractory to glucocorticoids and DMARDs (10, 16). The pathophysiological basis of sJIA is the activation of pro-inflammatory cytokines, especially IL-1β and IL-6 (17, 18). Currently, there are two main biological treatment strategies for sJIA: IL-1 and IL-6 biologic blockade (19, 20). However, in China, IL-1 blockers are still unavailable so that IL-6 blockers are the main biologic treatment for sJIA.

In our study, we found that fever, active arthritis, sJADAS-27 score, white blood cell count, ESR, and CRP can be relieved after 2 weeks of TCZ treatment. TCZ has significant short-term efficacy for sJIA. Furthermore, we observed sJIA patients with TCZ administration in early stage and found that it had better remission of active arthritis than those administrated in the late stage after 12 weeks of treatment. TCZ can quickly relieve fever and inflammation (indicated by decreased CRP and ESR) (21–24), especially for patients showing less active arthritis in the early stage. Doaa et al. (25) identified that younger patients with shorter disease duration and greater systemic manifestations showed more favorable outcomes by TCZ therapy. These observations are consistent with the window of opportunity hypothesis, which suggests that IL-6 blockade may be more effective in early sJIA, when the disease is characterized by more prominent systemic presentation and less active arthritis (26). Likewise, Alexeeva et al. (27) found that only earlier age at initiation of TCZ therapy was a statistically significant factor associated with reaching the best response to therapy in polyarticular JIA.

The course of sJIA varies and is divided into three phenotypes (7). In this study, sJIA was classified as monocyclic (*n* = 12, 15.6%), polycyclic (*n* = 14, 18.2%), and persistent course (*n* = 51, 66.2%). Bielak et al. (28) defined three phenotypes of sJIA: monocyclic, polycyclic, and polyarticular disease, which occurs as arthritis flares in >4 joints. Systemic inflammation, such as fever, elevated CRP, and ESR, is not more prominent in patients with multi-joint sJIA than single-joint sJIA, and systemic inflammation gradually evolves into an autoimmune disease phenotype (24, 29). The heterogeneity of sJIA contributes to the differences. Bielak et al. found that polycyclic and monocyclic sJIA responded better to tocilizumab than polyarticular sJIA. In our study, the clinical response was worst in patients with persistent course and the best in monocyclic. A previous study indicated that IL-1 inhibitors may be useful if patients do not respond to TCZ. sJIA with persistent course often show polyarticular JIA, and in these patients, TCZ is effective for fever and inflammation, but not for polyarthritis. Therefore, sJIA with persistent course who do not respond to TCZ may need an IL-1 antagonist or a TNF-a monoclonal antibody (30). To obtain optimal therapeutic responses, it is necessary to predict the disease phenotype of sJIA at an early stage. Singh-Grewal et al. (7) proposed that the clinical features observed at 3 months (the presence of active arthritis and fever) and 6 months (elevated ESR > 26 mm/H requiring corticosteroid treatment) are accurate predictors of a patient's disease course. This information helps identify patient's risk of developing into persistent disease and having a higher likelihood of a poor functional outcome, which need timely therapeutic intervention to prevent from joint damage and disability. Therefore, early TCZ treatment is recommended for such patients with early predictions that may be persistent courses.

The most common adverse effect observed in our study was granulocytopenia, which was mild and not accompanied by severe infection. Clinical remission was achieved in six of the seven cases, suggesting that patients who develop granulocytopenia after TCZ treatment may have a greater chance to obtain clinical remission. Neutropenia associated with sJIA is dependent on IL-6 levels, and leukocytopenia and granulocytopenia may be used as biomarkers of susceptibility to treatment with IL-6 monoclonal antibodies (31). There were two cases of MAS, which was not unexpected given that previous studies report that 20% to 25% of patients with sJIA treated with biological agents develop MAS (32). Current research suggests that TCZ does not prevent the occurrence of MAS, and indeed in some patients, TCZ may even trigger this reaction. The specific cause and mechanism are unclear. It is possible that when a patient experiences an obvious episode of active sJIA, TCZ antagonizes IL-6 which could trigger a negative feedback loop, therefore causing amplified and excessive inflammation and inducing MAS. Therefore, the timing of TCZ treatment is a critical factor.

## Limitation Points in This Study

In this study, glucocorticoids were standardized to ≤1 mg/kg/day to avoid the effect of high doses, but almost 60%–70% patients enrolled were taken at baseline. In addition, we did not study the beneficial effects of TCZ on glucocorticoid reduction and catch-up growth. Moreover, due to economic conditions, TCZ was not added every 2 weeks strictly, which could have a partial impact on the research results. It was a single-center retrospective study with low number of patients enrolled, so prospective cohort studies and further multicenter clinical studies are needed to shed additional light on this matter and ultimately improve the care of patients with sJIA.

## Conclusion

We demonstrated that TCZ can quickly relieve fever and inflammation, especially when patients have less active arthritis with shorter disease duration. The long-term efficacy of TCZ is related to the phenotype; therefore, it is necessary to predict the disease phenotype of sJIA at an early stage. These findings may help to define the profile of patients with sJIA who are more likely to benefit from TCZ.

## Data Availability Statement

The original contributions presented in the study are included in the article/supplementary material, further inquiries can be directed to the corresponding authors.

## Ethics Statement

Written informed consent was obtained from the minor(s)' legal guardian/next of kin for the publication of any potentially identifiable images or data included in this article.

## Author Contributions

XY analyzed the data and wrote the paper. WT and ZZ collected and analyzed the data. YZ and CL collected the patients. XT ideated the study and revised the paper. All authors approved the final version of the manuscript.

## Funding

This work was supported by the Natural Science Foundation of China (Grant Number 82001655).

## Conflict of Interest

The authors declare that the research was conducted in the absence of any commercial or financial relationships that could be construed as a potential conflict of interest.

## Publisher's Note

All claims expressed in this article are solely those of the authors and do not necessarily represent those of their affiliated organizations, or those of the publisher, the editors and the reviewers. Any product that may be evaluated in this article, or claim that may be made by its manufacturer, is not guaranteed or endorsed by the publisher.
